# Antioxidant Potential-Rich Betel Leaves (*Piper betle* L.) Exert Depigmenting Action by Triggering Autophagy and Downregulating MITF/Tyrosinase In Vitro and In Vivo

**DOI:** 10.3390/antiox12020374

**Published:** 2023-02-03

**Authors:** Md Badrul Alam, Na Hyun Park, Bo-Rim Song, Sang-Han Lee

**Affiliations:** 1 Department of Food Science and Biotechnology, Graduate School, Kyungpook National University, Daegu 41566, Republic of Korea; 2 Food and Bio-Industry Research Institute, Inner Beauty/Antiaging Center, Kyungpook National University, Daegu 41566, Republic of Korea

**Keywords:** antioxidant, autophagy, cAMP, melanogenesis, *Piper betle*

## Abstract

Each individual has a unique skin tone based on the types and quantities of melanin pigment, and oxidative stress is a key element in melanogenesis regulation. This research sought to understand the in vitro and in vivo antioxidant and depigmenting properties of betel leaves (*Piper betle* L.) extract (PBL) and the underlying mechanism. Ethyl acetate fractions of PBL (PBLA) demonstrated excellent phenolic content (342 ± 4.02 mgGAE/g) and strong DPPH, ABTS radicals, and nitric oxide (NO) scavenging activity with an IC_50_ value of 41.52 ± 1.02 μg/mL, 45.60 ± 0.56 μg/mL, and 51.42 ± 1.25 μg/mL, respectively. Contrarily, ethanolic extract of PBL (PBLE) showed potent mushroom, mice, and human tyrosinase inhibition activity (IC_50_ = 7.72 ± 0.98 μg/mL, 20.59 ± 0.83 μg/mL and 24.78 ± 0.56 μg/mL, respectively). According to gas chromatography–mass spectrometry, PBL is abundant in caryophyllene, eugenol, *O*-eugenol, 3-Allyl-6-methoxyphenyl acetate, and chavicol. An in vitro and in vivo investigation showed that PBLE suppressed tyrosinase (Tyr), tyrosinase-related protein-1 and -2 (Trp-1 and Trp-2), and microphthalmia-associated transcription factors (MITF), decreasing the formation of melanin in contrast to the untreated control. PBLE reduced the cyclic adenosine monophosphate (cAMP) response to an element-binding protein (CREB) phosphorylation by preventing the synthesis of cAMP. Additionally, it activates c-Jun N-terminal kinase (JNK) and p38 mitogen-activated protein kinases (p38), destroying Tyr and MITF and avoiding melanin production. Higher levels of microtubule-associated protein-light chain 3 (LC3-II), autophagy-related protein 5 (Atg5), Beclin 1, and lower levels of p62 demonstrate that PBLE exhibits significant anti-melanogenic effects via an autophagy-induction mechanism, both in vitro and in vivo. Additionally, PBLE significantly reduced the amount of lipid peroxidation while increasing the activity of several antioxidant enzymes in vivo, such as catalase, glutathione, superoxide dismutase, and thioredoxin. PBLE can therefore be employed in topical formulations as a potent skin-whitening agent.

## 1. Introduction

Melanin is synthesized from the precursor molecule L-tyrosine and is stored in subcellular organelles called “melanosomes” [1]. Melanogenesis is the complex process of melanin synthesis that involves both keratinocytes and melanocytes. Melanin is produced by melanocytes and is distributed to the surrounding keratinocytes in the skin. Melanosome formation and degeneration hence controls skin color [2]. Moreover, facultative pigmentation factors such as ultraviolet radiation (UVR), free radicals, and inflammation can have an impact on variations in skin color [3]. The body’s melanogenesis process serves multiple purposes. It aids in defending the skin against hazardous substances, UVR, and medicines [3]. However, excessive synthesis and accumulation of melanin in the skin can result in a number of cosmetically undesirable side effects, including freckles, chloasma, dermatitis, and geriatric skin pigmentation [4]. Furthermore, vitiligo, a depigmentation illness brought on by melanocytes lose, is not only a beautifying issue but also a significant societal issue [5]. Thus, the regulation of melanogenesis (melanin production) in the human epidermis is a unique clinical and scientific challenge.

Reactive oxygen species (ROS) and lipid peroxidation are overproduced when the skin is continuously subjected to endogenous and external oxidative stress [6]. ROS is known to have a significant impact on skin pigmentation. in vitro and in vivo experiments have demonstrated that the spontaneous polymerization of quinonic melanogenic intermediates produces H_2_O_2_ [7]. In addition, UVR increases the levels of ROS in keratinocytes and melanocytes, causes DNA damage, and encourages p53 activation, which controls the synthesis and activity of tyrosinase (Tyr) and tyrosinase-related protein-1 (Trp-1) [8]. In addition, there are several extracellular stimuli including α-melanocyte-stimulating hormone (α-MSH), and drugs that raise cyclic adenosine monophosphate (cAMP), such as forskolin and 3-isobutyl-1-methylxanthine (IBMX), that can also cause melanogenesis. IBMX and other methylxanthines promote melanogenesis through phosphodiesterase inhibition and cAMP augmentation. IBMX-activated cAMP phosphorylates the phosphoinositide 3-kinases/protein kinase B (PI3K/Akt), which controls the cAMP response element-binding protein (CREB), resulting in the activation of the microphthalmia-associated transcription factor (MITF) in melanogenesis [9]. Various kinase proteins including p38, c-jun N-terminal kinases (JNKs), and extracellular signal-regulated protein kinases (ERKs), also control melanogenesis [4].

According to a prior study, faulty “autophagy” systems may lead to skin pigmentation problems. In order to maintain cellular homeostasis, autophagy recycles and eliminates defective proteins and organelles in a conserved manner [10]. Autophagy was formerly thought to be a non-selective breakdown pathway; however, new research demonstrates that it can be selective for certain organelles, including mitophagy, pexophagy, and xenophagy [11]. A distinct collection of proteins regulates autophagy. Autophagy requires the transition of the microtubule-associated protein-light chain 3 (LC3 I: the cytosolic form of LC3, into LC3 II: the lipidated form), which also produces “autophagosomes” by connecting with distinct membranes. The proteins LC3 II and p62 collaborate to facilitate selective autophagosome degradation. The LC3-II/LC3-I ratio is frequently used as an autophagy indication [12]. The link between skin tone and autophagy in diverse ethnic groups has been investigated. Caucasian melanocytes exhibit greater autophagy activity than African American melanocytes, while hyperpigmented skin, such as senile lentigo, exhibits lower autophagy [13]. It was understood that autophagy and its regulators play multiple functions in maintaining the homeostasis of melanosomes. In contrast to MITF regulators and Tyr inhibitors, autophagy may degrade already produced melanin [14]. Consequently, autophagy-mediated suppression of melanogenesis may be particularly useful therapeutic goals for a variety of skin disorders. Therefore, autophagy inducers can be viewed as crucial targets in the regulation of melanogenesis.

To treat pathological skin pigmentation and hyperpigmentation illnesses, various Tyr inhibitors have been developed. Effective Tyr inhibitors found in whitening products have severe drawbacks, including being highly cytotoxic and unstable in the presence of oxygen and water, which restrict their use [4]. Thus, natural components are currently being investigated in the study and development of safe and effective skin depigmenting agents in the cosmetics industry because of their low toxicity and favorable side effects profile. Betel leaves (*Piper betle* L.) are a root-climbing vine with heart-shaped leaves. They are a profitable crop for horticulture, recreation, and medicine. In Asian nations, they have long been utilized as a traditional herbal treatment. The most prized medical, religious, and ceremonial part of the plant is its leaves, which are used as a mouthwash because of its potent spicy, sweet, and aromatic flavor [15]. Betel leaves are incredibly nutrient-dense, including enzymes, vitamins, minerals, proteins, and advantageous bioactive substances for the remedy of the brain, liver, and cardiac diseases [16]. In addition, polyphenols, alkaloids, steroids, saponins, and tannins in betel leaves have antibacterial, anti-cariogenic, anti-fungal, anti-larval, antiprotozoal, anti-allergic, anti-helminthic, anti-filarial, antitumor, anti-diabetic, antibacterial, hypotensive, respiratory, and depressant effects, among others [17,18,19,20,21]. The demand for its goods, including herbal medications, treatments, and natural herbal formulations, has grown recently. The value-added products (powder, leaves, extract, and essential oil) made from betel leaves relieve headaches, arthritis, mental discomfort, bronchitis, asthma, and skin and eye disorders [22,23,24]. However, the potential benefits of betel leaves in dermatology have not yet been investigated. This study aims to further efforts to develop novel and beneficial cosmetic chemicals, dietary supplements, and functional foods by investigating the effects of betel leaves extract on oxidative stress and melanogenesis, in vitro and in vivo. In order to pinpoint the bioactive substances present in PBLE, gas chromatography–mass spectrophotometric analysis was also performed.

## 2. Materials and Methods

### 2.1. Plant Materials and Extraction

Fresh betel leaves (PBL) were harvested using a conventional agricultural method from a particular shade garden in Khulna, Bangladesh (24°31′18″ N and 88°43′27″ E). In February 2021, PBL of different sizes that were fresh, healthy, green, and fully grown were picked from over 200 plants and kept out of the sun to retain the aroma of leaves. An experienced researcher from Bangladesh’s National Herbarium taxonomically identified the plant parts (voucher specimen No. DACB 38091), which were preserved and stored in our lab. A total of 100 g of dried ground leaves were added to 1000 mL of ethanol and were thoroughly mixed with a stirrer. The heat extraction was performed using a flask with a circular bottom and a condenser to stop solvent evaporation in a temperature-controlled heater (Soxhlet water bath C-WBS-D6, Changshin Science, Seoul, Korea) at 50 °C for 1 h. Subsequently, the samples were filtered with Whatman No. 1 filter paper (Schleicher & Schuell, Keene, NH, USA) and dried in a rotary evaporator (Tokyo Rikakikai Co. Ltd., Tokyo, Japan) at 40 °C and 50 rpm to obtain an ethanolic extract of PBL (PBLE: 50.52 g). The resultant residue was suspended in 500 mL dH_2_O and progressively partitioned with hexane and ethyl acetate (each 500 mL × 3) to get a fraction of hexane (PBLH: 12.25 g), ethyl acetate (PBLA: 28.23 g), and aqueous (PBLW: 10.55 g). Extraction was performed in a trice, while each extraction had minimal light exposure and was stored at −20 °C before further investigation.

### 2.2. Gas-Chromatography Mass Spectrometric Analysis

The GC–MS study was carried out using an Agilent 7890B-5977B MSD system (Agilent Technologies, Santa Clara, CA, USA) with a PAL autosampler and DB-WAX capillary column (60 m × 250 m, 0.25 m film thickness). The sample was made using SPME fibers coated with CAR/DVB/PDMS (divinylbenzene/carboxen/polydimethyl-siloxane) and injected in split mode at 250 °C. The initial oven temperature was 40 °C for 2 min. First, 220 °C was elevated at 2 °C/min, then 240 °C at 20 °C/min, and then held for 10 min. The carrier gas was helium (flow rate: 1 mL/min). The mass spectrometry conditions were 230 °C, 1 min of solvent delay, a 35–400 m/z scanned mass range, and 230 °C quadrupole and 150 °C electron ionization ion source temperatures, respectively.

### 2.3. Antioxidant Activities

The total phenolic content (TPC) and total flavonoid content (TFC) of various extracts/fractions of PBL were measured using the Folin–Ciocalteu test and the aluminum chloride colorimetric method, respectively [25]. The TPC (y = 0.0502x + 0.0038; r^2^ = 0.9935) and TFC (y = 0.024x + 0.0051; r^2^ = 0.9894) was determined using the corresponding regression equations for the calibration curves. The TPC was expressed in terms of the gallic acid equivalent (mg)/dry weight sample (g) (mgGAE/g) and the TFC in terms of the catechin equivalent (mg)/dry weight sample (g) (mgCE/g).

The antioxidant capability of various extract/fractions of PBL was evaluated using the procedures outlined in earlier publications [25]. Antioxidant experiments employed ascorbic acid as a positive control. The percentage inhibition of 2,2-diphenyl-1-picrylhydrazyl- (DPPH), 2,2′-azino-bis(3-ethylbenzothiazoline-6-sulfonic acid- (ABTS), nitric oxide (NO), and hydroxyl radical (HO) scavenging was calculated using Equation (1).

(1) $$\left(\%\;inhibition\right)=\left[\left(1-\frac{Abs_{sample}}{Abs_{control}}\right)\right]\;\times \;100$$

where Abs_control_ and Abs_sample_ are the absorbance of the control and absorbance of the sample, respectively. Each sample was examined three times. Each sample’s 50% inhibitory concentration (IC_50_) value was also computed to compare various extract/fractions efficacy.

### 2.4. Cell Culture and Cell Viability Assay

Highly pigmented human melanoma cells (MNT-1), purchased from ATCC (Rockville, MD, USA), were kept alive at 37 °C under 5% CO_2_ in Dulbecco’s Modified Eagle Medium (DMEM), which was supplemented with 10% fetal bovine serum (FBS, Hyclone, Utah, UT, USA), streptomycin–penicillin (100 µg/mL each), 10% AIM-V medium, 0.1mM non-essential amino acid mix, and 1mM sodium pyruvate (Invitrogen, Waltham, MA, USA). To confirm the cytotoxicity of PBLE the 3-(4,5-dimethyl-2-thiazolyl)-2,5-diphenyl-2H-tetrazolium bromide (MTT) assay was used as described in our previous report [4].

### 2.5. Effect of PBLE on Mushroom, Mice, and Human Tyrosinase Activity

Cells (5 × 10^5^ cells/mL) were plated into a 24-well culture plate (BD Falcon, Bedford, MA, USA) and were allowed to grow overnight. The old media were replaced with new media, which was then treated with varying doses of PBLE (3, 10, and 30 μg/mL) or arbutin (10, 30, and 100 μg/mL). After 3 days, cells were washed with PBS, lysed using lysis buffer (1% tryptone in PBS, pH 6.8) at −4 °C and centrifuged at 10,000× *g* for 30 min at 4 °C to collect the supernatant as a source of tyrosinase (Tyr). A 96-well microplate (SPL, Pocheon, Korea) was first filled with phosphate buffer (100 mM; pH 6.5), with or without PBLE or arbutin. L-tyrosine (0.01 M) and mushroom tyrosinase (200 units/mL)/20 μg lysate were then added, and the mixture was incubated at 37 °C for 5 min. Dopachrome synthesis was measured at 490 nm, and the percentage inhibition was calculated using Equation (2).

(2) $$EA\;\left(\%\;inhibition\right)=\left[\left(1-\frac{Abs_{sample}}{Abs_{control}}\right)\right]\times \;100$$

where EA, Abs_control_, and Abs_sample_ are the enzyme activity, absorbance of the control, and absorbance of the sample, respectively. All samples were analyzed in triplicates [4,26].

### 2.6. Effect of PBLE on Melanin Content

Cells (5 × 10^5^ cells/mL) were plated into a 24-well culture plate (BD Falcon, Bedford, MA, USA) and allowed to grow overnight. The old media were replaced with new media, which was then treated with varying doses of PBLE (3, 10, and 30 μg/mL) or arbutin (100 μg/mL). After 3 days, cells were washed with PBS and lysed with 1 N NaOH. A microplate reader (Thermo Fisher Scientific, Finland) was used to measure the sample’s absorbance at 405 nm and the percentage of inhibition of melanin was calculated using Equation (3).

(3) $$Melanin\;production\;\left(\%\;inhibition\right)=\left[\left(\frac{A-B}{A}\right)\times \;100\right]$$

where A and B are the absorbance of cells lysate treated with and without PBLE or arbutin (positive control), respectively [4].

### 2.7. Zymography for Tyrosinase Activity Measurement

MNT-1 cells (1.5 × 10^5^ cells/mL) were cultured with varying doses of PBLE (3, 10, and 30 μg/mL) or arbutin (100 μg/mL) for 72 h. The cells were then rinsed with cold PBS and lysed with protease and phosphatase inhibitor containing RIPA buffer (Abcam, Cambridge, UK), followed by quantification of protein using bicinchoninic acid (BCA) protein assay kit (Pierce Biotechnology, Rockford, IL, USA). The protein (50 µg) was combined with 0.1 M sodium phosphate buffer (pH 6.8) and SDS loading buffer (Biosesang, Seongnam, Korea) and was separated using 10% SDS-polyacrylamide gel electrophoresis. The gel was stained with 5 mM L-DOPA at 37 °C for 2 h [4].

### 2.8. The Concentration of cAMP Determination

Cellular cAMP levels were determined using a cAMP direct immunoassay kit (Biovision, San Francisco, CA, USA). In 6-well plates, cells were cultured overnight followed by treatment with PBLE (3, 10, and 30 μg/mL) or arbutin (100 μg/mL). The cells were then lysed and harvested using 1N HCl. Subsequently, the reaction was stopped, and cAMP content was measured according to the manufacturer’s instruction.

### 2.9. Animal Experiments

Six-week-old male hairless mice (HRM-2) were bought from Central Lab Animals, Inc. (Seoul, Korea). They were kept in a climate-controlled animal room with a 12 h light/12 h dark cycle, a 23 ± 1 °C temperature, a 55 ± 5% humidity level, free access to water, and a typical laboratory meal. The animals were permissible to adjust for a week. They were aimlessly allocated into five groups (five mice each). All animal studies were carried out in accordance with the institutional animal care and used the committee of Kyungpook National University-approved guidelines for animal experiments (KNU-2021-0025). A mixture of propylene glycol and ethanol (3:7) was used to dissolve PBLEL (10 mg/Kg), PBLEH (30 mg/Kg), and arbutin (300 mg/Kg). All samples were topically applied once daily during the study period to a designated 3 cm × 3 cm area on the dorsal skin of the hairless mice (Supplementary Materials Figure S1). Mice were expressly pre-treated for 3 days with the sample or control solution before exposure to UVB (302 nm; 150 mJ/cm^2^). In a UVB exposure chamber, mice were subjected to UVB radiation for 10 min every other day from day 4 to day 28, 30 min after receiving either the sample or control solution. On day 29, mice were euthanized, the dorsal skin was taken off and frozen using liquid nitrogen for immunoblotting assay, and the excised skin was preserved in a 4% paraformaldehyde solution before staining. The color of the treated skin areas was evaluated using a CR-10 spectrophotometer (Konica Minolta Sensing, Inc., Sakai, Osaka, Japan) on day 29. According to the International Commission de l’Eclairage color standard, L* was used to characterize the color, with higher and lower numbers signifying whiter and blacker colors, respectively. In addition, the development of melanin in the skin of hairless mice was examined using the Fontana–Masson staining method using a commercial kit (American Mastertech, Inc., Lodi, CA, USA). The melanin spots were visualized by an AE-31 light microscope (Motic, Hong Kong).

### 2.10. Antioxidant Enzyme Assays

PBLEs effects on the activity of the endogenous antioxidant enzymes, such as superoxide dismutase (SOD), catalase (CAT), glutathione (GSH), and thioredoxin (Trx), were assessed using the appropriate assay kits.

SOD is a critical player in the antioxidant defense mechanism in cells that converts the superoxide anion to molecular oxygen and hydrogen peroxide. Superoxide radicals produced by xanthine oxidase and hypoxanthine are detected using the SOD assay kit (Cayman Chemical, catalog no. 706002, Ann Arbor, MI, USA), and SOD activity was expressed as U/mg protein.

CAT detoxifies the cell-toxic hydrogen peroxide. The activity of CAT was measured using a CAT assay kit (Cayman Chemical, catalog no. 707002, Ann Arbor, MI, USA), following manufacturer instructions, and was expressed as U/mg protein.

The GSH assay kit (Cayman Chemical, catalog no. 703002, Ann Arbor, MI, USA) allows for the measurement of total GSH. GSH produces the yellow 5-thio-2-nitrobenzoic acid (TNB) as a byproduct of the reaction with 5,5′-dithio-bis-2-nitrobenzoic acid (DTNB). The rate of TNB synthesis is directly proportional to the GSH formation, which is quantified at 405–414 nm and represented as mol/mg protein.

For thioredoxin (Trx) activity, ELISA kit (MyBioSource, Inc., catalog No: MBS2019144, San Diego, CA, USA) was used, according to the manufacturer’s instructions.

### 2.11. Measurement of Lipid Peroxidation (LPO)

To quantify the oxidative change in biological materials, malondialdehyde (MDA), a byproduct of LPO, was measured spectrophotometrically using a thiobarbituric acid (TBA) assay [27]. Briefly, 125 µL of tissue homogenate was mixed with 250 µL of 15% TCA and 250 µL of 0.37% TBA, vortexed, and incubated at 100 °C for 10 min. The mixtures were subsequently centrifuged at 12,000× *g* for 5 min at 4 °C and the absorbance was measured at 535 nm. Concentration of MDA in homogenates was calculated using a standard curve prepared with 25 µM 1,1,3,3-Tetraethoxypropane (TEP) and was expressed in nanomoles of MDA per mg tissue.

### 2.12. Preparation of Cell Lysates and Western Blotting

Sodium dodecyl sulfate (SDS) buffer (3 M, Maplewood, Minnesota, USA) was added to the cell lysates, and the mixture was then denatured at 100 °C for 5 min [26]. A sufficient amount of proteins (30 µg) were electrophoretically separated on a 10% SDS-polyacrylamide gel and blotted onto nitrocellulose membranes (Whatman, Dassel, Germany). Prior to being treated with primary antibodies (Supplementary Table S1), the membranes were blocked with 5% skim milk in TBST buffer. Anti-mouse IgG-horseradish peroxidase (HRP) and anti-goat IgG-HRP (Bioworld Technology, St. Louis Park, MN, USA) were used as secondary antibodies. An ECL solution system (Perkin Elmer) was used to detect the antigen–antibody reaction.

### 2.13. Statistical Analysis

The mean and standard deviation (n = 3) were used to express the data. Using SPSS for Windows version 10.07 (Chicago, IL, USA), a one-way analysis of variance and Tukey’s multiple comparison test were conducted, and *p* < 0.05 were considered statistically significant. SigmaPlot software (SigmaPlot, Ver 12.5, Systat Software, Inc., Chicago, IL, USA) was used to draw the graph. Principle component analysis (PCA) was performed utilized to analyze the effect of solvent on TPC, TFC, and antioxidant activity, and also to learn the correlations between these variables. PCA was carried out using Minitab Statistical Software (Version 18.0, Minitab Inc., Enterprise Drive State College, PA, USA).

## 3. Results

### 3.1. Gas Chromatography–Mass Spectrometry (GC–MS) Analysis of PBLE

GC–MS is the most effective method for identifying the bioactive components of plant species, such as acids, alcohols, esters, long-chain hydrocarbons, alkaloids, steroids, amino, and nitro chemicals. Consequently, gas chromatography–mass spectrometry (GC–MS) in conjunction with specific detection techniques have evolved into sophisticated methods for analyzing diverse substances with medical values [28]. The GC–MS analysis of PBL indicated the presence of 36 bioactive compounds, which were identified by comparing their peak retention time, peak area (%), and mass spectrum fragmentation patterns to those of known compounds documented in the National Institute of Standards and Technology (NIST) library. Table 1 presents the compounds with their retention time (RT), molecular formula, molecular weight, and area (%).

The most abundant bioactive compounds were *O*-eugenol (RT-77.68; 0.30–51.47%), *O*-eugenol acetate (RT-80.62; 0.20–26.72%), cavicol (RT-83.94; 0.25–3.96%), caryophyllene (RT-46.81; 0.03–1.80%), copaene (RT-40.47; 0.01–1.84%), α-humulene (RT-50.99; 0.07–1.59%), methyleugenol (RT-69.12; 0.08–2.54%), eugenol (RT-76.39; 0.27–9.73%), and (-) spathulenol (RT-74.56; 0.51%). PBL is mostly composed of phenyl propanoids, sesquiterpene, hydrocarbons, and their low-molecular-weight derivatives. PBL contained sesquiterpene hydrocarbons such as β-bourbonene (0.03–0.74%), trans-α-bergamotene (0.09–2.19%), germacrene D (0.09–1.19%), β-bisabolene (0.26–1.26%), and monoterpene hydrocarbons such as cubebene (0.01–2.81%), and α-murolene (0.02–1.05%). It has been claimed that the main ingredients in Tamluk mitha variety betel leaves are chavibetol, estragole, β-cubebene, chavicol, and caryophyllene [28]. Notably, there are a number of variables, including plant type, development stage, harvesting period, meteorological conditions, nation of origin, ecological setting, and geographic location, that affect how different PBLs differ in their chemical content.

### 3.2. Total Phenol, Total Flavonoid, and Antioxidant Activities of PBL

**Figure 1:**
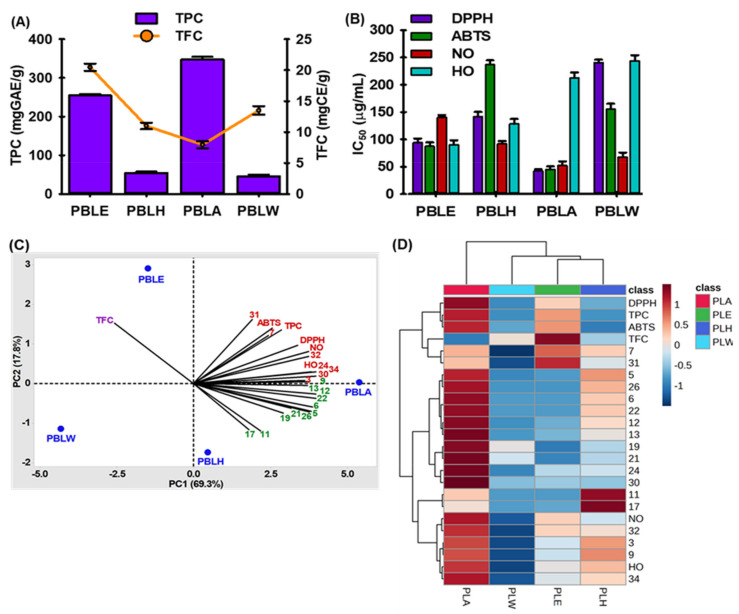
Antioxidant effects of extract/factions of betel leaves extract (PBL). (**A**) Total phenol (TPC) and flavonoid contents (TFC),. (**B**) Radical scavenging activities of PBL extract/fractions. (**C**) Principal component analysis. (**D**) Hierarchical clustering analysis with heat map. mgGAE/g: mg gallic acid equivalent/g extract; mgCE/g: mg catechin equivalent/g extract; PBLE: ethanolic extract of PBL; PBLH: hexane fraction of PBL; PBLA: ethyl acetate fraction of PBL; and PBLW: aqueous fraction of PBL. Numerical no. represents the compounds described in Table 1.

Phenolic compounds are crucial components of plants and have redox characteristics that contribute to their antioxidant action [29]. As shown in Figure 1A, the ethyl acetate fraction of PBL (PBLA) had the highest phenolic content (343.58 ± 1.53 mgGAE/g) and decreased in the following order: ethanolic extract of PBL (PBLE) > hexane fraction of PBL (PBLH) > aqueous fraction of PBL (PBLW). Compared to the literature, this investigation’s phenolic content differed little [30]. Biological and environmental variables influence a plant’s phenolic concentrations. Their solubility is further controlled by the level of polymerization of phenolic compounds, the interactions between them, and the type of extraction and solvent used [30].

The abundance of flavonoid-containing plants may act as beneficial sources of antioxidants that help to improve an organism’s total antioxidant capacity and provide protection against lipid peroxidation [29]. The total flavonoids content ranged from 7.96 ± 0.56 to 20.47 ± 0.58 (Figure 1A). Compared to PBLA, PBLE has a higher flavonoid than phenolic content.

As shown in Figure 1B, different extract/fractions of PBL showed remarkable scavenging activities of 2, 2-diphenyl 1-picrylhydrazyl- (DPPH), 2, 2′-azino-bis (3-ethylbenzothiazoline-6-sulfonic acid- (ABTS), nitric oxide-(NO), and hydroxyl-(HO) radical. PBLA showed the highest DPPH, ABTS, and NO scavenging activity with IC_50_ values of 40.59 ± 0.58, 42.52 ± 1.58, and 48.56 ± 1.28 μg/mL, respectively. In contrast, PBLE showed the highest HO-radical scavenging activity (IC_50_ = 97.52 ± 0.88 μg/mL).

### 3.3. Chemometric Analysis of PBL Extract/Fractions

Only 18 of the 36 chemicals (cut-off value of 1% was present in each sample) were chosen before chemometric analysis (Table 1). Finally, 24 relevant variables for PCA, including 18 chemicals and 6 antioxidant characteristics, were chosen. A principal component analysis (PCA) statistical technique enables the direct modeling of various sample datasets and a better understanding of their relationships. Therefore, the interdependence of variables (chemical compositions and biological impacts) was analyzed in this study, using PCA to pinpoint the most distinctive variables within the datasets. It overviews the correlations between the variables and their capacity to distinguish between the four ex-tracts (PBLE, PBLH, PBLA, and PBLW). The PBL extract datasets produced two principal components (PCs). The variance was explained by PC1 (69.3%) and PC2 (17.8%), with a combined percentage of total variations of 87.1%. Biplots were used to display the PCA results (Figure 1C). As shown in Figure 1C, compounds 3, 7, 24, 30-32, 36, TPC, DPPH, ABTS, NO, and the HO-radical scavenging activity contributed positively to both PC1 and PC2. TFC had a positive contribution to PC2 while compounds 5, 6, 9, 11-13, 17, 19, 21, 22, and 26 had positive contributions to PC1 but negative contributions to PC2.

The PBL samples were clustered into three categories that were compared to the dendrogram from the hierarchical clustering analysis (HCA) and supported the PCA result (Figure 1D). Compared to PBLW and PBLA, PBLE and PBLH were more closely related (Figure 1D). The heatmap displays the contribution of significant variables (Figure 1D). The majority of the compounds were seen to be significant and plentiful in PBLA fractions. In contrast, compounds 11 and 17 were relatively abundant in PBLH, while compound 31 was high and distinct for PBLE (Figure 1D).

### 3.4. Effect of PBLE on Tyrosinase (Tyr) Activity

**Figure 2:**
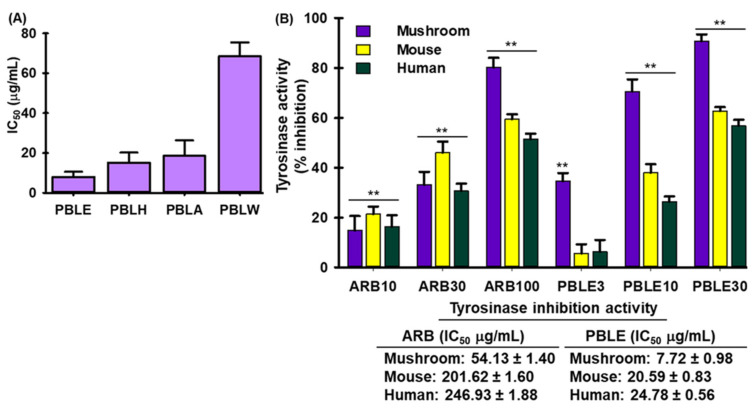
Tyrosinase inhibitory effects of PBL extract/fractions. (**A**) Effect of PBL extract/fractions on mushroom tyrosinase. (**B**) The inhibitory effect of PBLE on mushroom tyrosinase, mice, and human cellular tyrosinase. Mean ± SD (n = 3). ** *p* < 0.05 vs. non-treated (NT) control. PBLE: ethanolic extract of PBL; PBLH: hexane fraction of PBL; PBLA: ethyl acetate fraction of PBL; PBLW: aqueous fraction of PBL; and ARB: arbutin.

Melanogenesis forms the skin pigment melanin. The most prevalent method for inhibiting melanogenesis is to decrease tyrosinase (Tyr) activity. PBLE showed the highest inhibitory activity towards the mushroom tyrosinase enzyme followed by PBLH, PBLA, and PBLW (Figure 2A). Thus, PBLE extracts were selected for further research. PBLE inhibited tyrosinase activity in mushroom, mice, and humans in a concentration-dependent manner, with IC_50_ values of 7.72 ± 0.98 μg/mL, 20.59 ± 0.83 μg/mL, and 24.78 ± 0.56 μg/mL, respectively, while arbutin (ARB) had IC_50_ values of 54.13 ± 1.40 μg/mL, 201.62 ± 1.60 μg/mL, and 246.93 ± 1.88 μg/mL, respectively (Figure 2B). These findings suggested that PBLE has a powerful ability to decrease melanogenesis. Furthermore, to ascertain the kinetic parameter, an investigation on PBLEs effects on monophenolase-activated Tyr was also conducted. Following the onset of tyrosinase’s enzymatic reaction, there is a discernible lag period in the monophenolase activity [31]. The monophenolase activated form of Tyr was markedly inhibited by PBLE in a concentration-dependent manner, and more significantly, PBLE (30 μg/mL) widened the lag phase by 15 min in comparison to the control (Supplementary Figure S2).

### 3.5. Depigmenting Effect of PBLE on Both Hyperpigmented Melanocyte Cells

**Figure 3:**
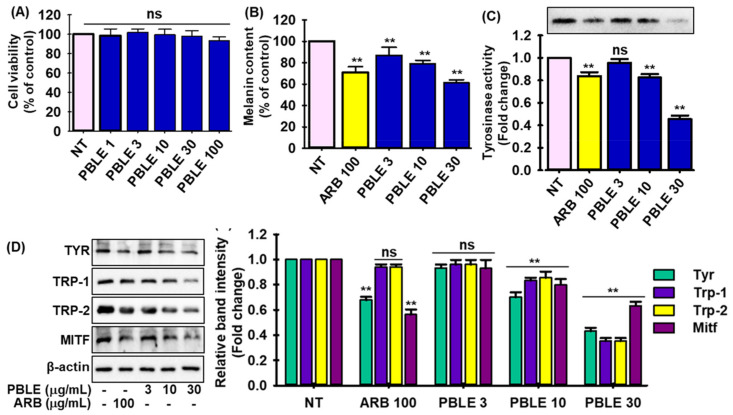
The depigmenting effects of PBLE on MNT-1 cells. Effect of PBLE on (**A**) cell viability, (**B**) melanin production, (**C**) Tyrosinase (Tyr) inhibition activity measured by zymography, (**D**) proteins involved in melanogenesis, including microphthalmia-associated transcription factor (MITF), Tyr, tyrosinase-related protein-1 (Trp-1), and -2 (Trp-2), as determined by Western blotting. Densitometric analysis of relative protein expression matching to Figure 3D. Mean ± SD (n = 3). ** *p* < 0.05 vs. non-treated (NT) control. NS: non-significant compared to NT. ARB: arbutin.

In order to determine the optimal experimental dose of PBLE, MNT-1 cells were exposed to varying concentrations of PBLE (1 to 100 µg/mL), and the effect of PBLE on cell viability was evaluated. As shown in Figure 3A, PBLE exhibited no cytotoxic effects up to 100 µg/mL. PBLE significantly decreased melanin synthesis in a concentration-dependent manner (Figure 3B, 3rd to 5th column). Curiously, PBLE at higher doses inhibited melanin production by 41.25%, whereas ARB did so by 29.56% (Figure 3B). In addition, L-DOPA zymography was conducted to determine the impact of PBLE on intracellular Tyr activity in the cells. As shown in Figure 3C, PBLE (30 μg/mL) treatment significantly decreased cellular Tyr activity 2-fold in MNT-1 cells, respectively, while ARB (100 μg/mL) inhibited 0.5-fold.

Additionally, immunoblotting was used to examine the effects of PBLE (3, 10, and 30 μg/mL) on the expression of melanogenesis-related proteins such as Tyr, tyrosinase-related proteins (Trp-1, and -2), and the microphthalmia-associated transcription factor (MITF) in MNT-1 cells. As demonstrated in Figure 3D, the protein expression of Tyr, Trp-1, Trp-2, and MITF was strongly repressed by PBLE in a concentration-dependent fashion compared to the untreated control. Notably, PBLE (30 μg/mL) showed stronger melanogenesis biomarker downregulation effects than ARB. The current findings imply that PBLE inhibits melanin biosynthesis by attenuating MITF and its downstream proteins’ expression.

### 3.6. Effect of PBLE on the Melanogenesis-Related Signaling

**Figure 4:**
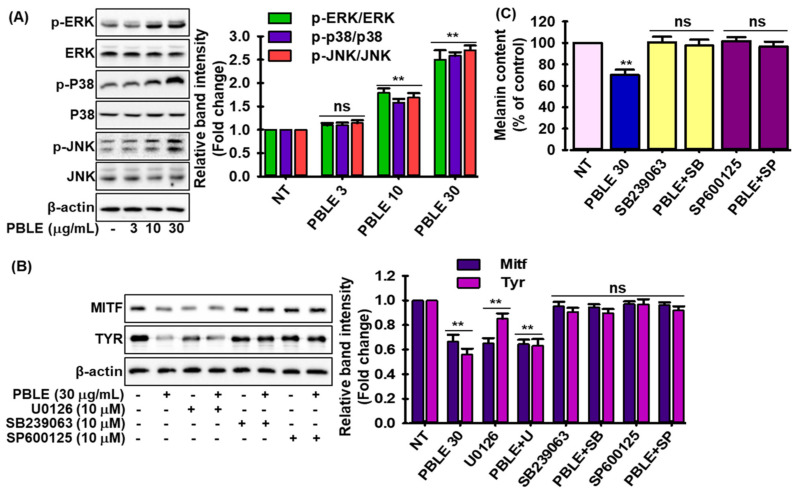
The effect of PBLE on mitogen-activated protein kinase (MAPK) phosphorylation in MNT-1 cells. (**A**) Effects of PBLE concentration on the activation of MAPK proteins. (**B**) Effects of various MAPK protein inhibitors on the expression of MITF and Tyr, with or without PBLE. Densitometric analysis of relative protein expression are presented in adjacent figure (**C**) Effects of different MAPK protein inhibitors on melanin production, with or without PBLE. Mean ± SD (n = 3). ** *p* < 0.05 against the untreated (NT) control. NS: non-significant compared to equivalent inhibitor alone. U0126: extracellular signal-regulated protein kinases (ERKs) inhibitor; SB239063: p38 inhibitor; and SP600125: c-jun N-terminal kinases (JNKs) inhibitor.

The MNT-1 cells were exposed to PBLE to better understand its melanogenic mechanism. Then, mitogen-activated protein kinase (MAPK) protein expression was determined by Western blot. As shown in Figure 4A, after 30 min of treatment, PBLE led to the dose-dependent phosphorylation of MAPKs (p38, c-jun N-terminal kinases (JNKs), and extracellular signal-regulated protein kinases (ERKs)). These findings imply that MAPK signaling may be involved in PBLE’s melanogenesis suppression. Furthermore, cells were pretreated with selective inhibitors of the p38 (SB209190), JNKs (SP600125), or ERKs (U0126) before PBLE administration to ascertain whether elevated phosphorylation of MAPKs were responsible for the inhibition of MITF and Tyr expression. Treatment with p38 and JNK inhibitors abolished the anti-melanogenic effects of PBLE via MITF and Tyr expression, while ERK inhibitors failed to reverse the PBLE activity (Figure 4B). To further explore the effects of PBLE-induced phosphorylation of MAPKs on melanin synthesis, the melanin levels in PBLE-treated cells were assessed in the presence of MAPK inhibitors. As anticipated, p38 and JNK inhibitors prevented PBLE’s downregulation of melanin (Figure 4C). These findings suggest that the PBLE-induced attenuation of melanogenesis involves the activation of the p38 and JNK pathways.

### 3.7. Effects of PBLE on IBMX (Isobutylmethylxanthine)-Induced Melanogenesis

**Figure 5:**
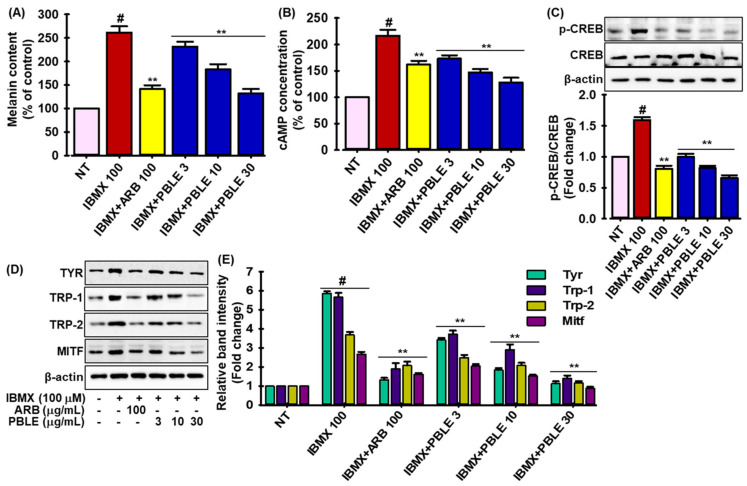
The depigmenting impact of PBLE on isobutylmethylxanthine (IBMX)-induced MNT-1 cells. (**A**) PBLE prevents the overproduction of melanin caused by IBMX. (**B**) The effect of PBLE on IBMX-induced cellular cyclic adenosine monophosphate (cAMP) production. (**C**) PBLE inhibits IBMX-stimulated causes cAMP response to element-binding protein (CREB) protein phosphorylation. Densitometric analysis of relative protein expression are presented in adjacent figure. (**D**) Effect of PBLE on proteins associated in melanogenesis, such as MITF, Tyr, Trp-1, and Trp-2, in IBMX-stimulated cells, as measured by Western blotting. (**E**) Densitometric analysis of relative protein expression, as depicted in Figure 5D. Mean ± SD (n = 3). # *p* < 0.05 vs. non-treated (NT) control. ** *p* < 0.05 vs. IBMX-treated control. ARB: arbutin.

In addition, the effects of PBLE on isobutylmethylxanthine (IBMX)-induced melanogenesis in MNT-1 cells were studied. IBMX treatment dramatically enhanced the melanin synthesis by almost 2.5 times in MNT-1 cells compared to untreated cells, whereas PBLE treatment significantly and concentration-dependently reversed this tendency in MNT-1 cells (Figure 5A), and at a higher dose (30 μg/mL), PBLE showed a stronger activity than ARB. IBMX increases intracellular cyclic adenosine monophosphate (cAMP) levels by blocking cAMP phosphodiesterase, which causes the cAMP response element-binding protein (CREB) to become phosphorylated and control MITF [32]. Thus, the effects of PBLE on the production of cellular cAMP and the phosphorylation of CREB were also assessed in IBMX-induced MNT-1 cells. As expected, IBMX treatment dramatically increased the cellular cAMP in MNT-1 cells, whereas a PBLE injection markedly decreased the cellular cAMP content in a dose-dependent manner and unlike ARB, higher doses of PBLE had strong cAMP-suppressing action (Figure 5B). Additionally, IBMX increased the phosphorylation of CREB in MNT-1 cells by 1.6-fold, but PBLE treatment significantly reversed this tendency in a dose-dependent manner (Figure 5C). It is noteworthy that PBLE demonstrated greater activity than ARB. The effect of PBLE on IBMX-induced overexpression of the melanogenesis marker was next examined by the Western blot analysis. As seen in Figure 5D,E, IBMX insult increased Tyr, Trp-1, Trp-2, and MITF expression, but PBLE therapy successfully reversed this trend in a dose-dependent manner. Interestingly, higher doses of PBLE, as opposed to ARB, strongly downregulated the melanogenesis biomarkers. This result suggests that PBLE is also able to decrease melanin synthesis by inhibiting the cAMP/CREB signaling cascade.

### 3.8. Regulation of Autophagy by PBLE

**Figure 6:**
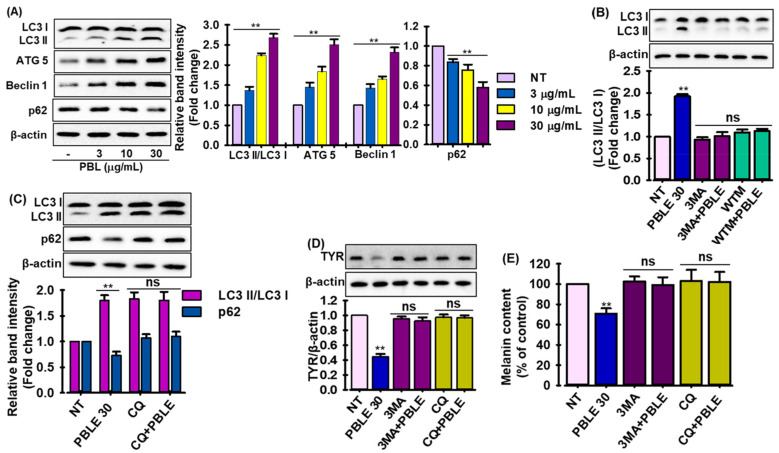
The effect of PBLE on the activation of autophagy in MNT-1 cells. (**A**) Effect of PBLE on proteins associated with autophagy, such as microtubule-associated protein-light chain 3 (LC3), Atg5, Beclin 1, and p62, in MNT-1 cells, as measured by Western blotting. (**B**) Effects of autophagy inhibitors on the expression of LC3, with or without PBLE. (**C**) Effects of autophagosome fusion with lysosome inhibitor on the expression of LC3 and p62, with or without PBLE. (**D**) Effects of autophagy inhibitors on the expression of Tyr, with or without PBLE. (**E**) Effects of autophagy inhibitors on the melanin production, with or without PBLE. Mean ± SD (n = 3). ** *p* < 0.05 against the untreated (NT) control. NS: non-significant compared to equivalent inhibitor alone. 3MA: 3-Methyladenine (autophagy inhibitor), WTM: wortmannin, (PI3K inhibitor), and CQ: chloroquine (autophagosome fusion inhibitor).

Recent research has suggested that autophagy is crucial for melanogenesis [33]. To determine whether PBLE has the ability to regulate autophagy, several autophagy markers, including microtubule-associated protein-light chain 3 (LC3) II, Atg5, Beclin 1, and p62, were tested by immunoblotting in MNT-1 cells. As demonstrated in Figure 6A, PBLE treatment enhanced Atg5 and Beclin 1 protein expression as well as the conversion of LC3 I to LC3 II, while increasing p62 protein degradation in a concentration-dependent manner. This indicates that PBLE not only effectively stimulated the development of autophagosomes, but also had a significant role in the autophagic flux in MNT-1 cells. 3-Methyladenine (3-MA), an autophagy inhibitor, and wortmannin, a PI3K-AKT inhibitor, were used to assess the conversion of LC3 I to LC3 II in MNT-1 cells to confirm the effects of PBLE on autophagosome production. As depicted in Figure 6B, PBLE can promote autophagy in MNT-1 cells since the conversion of LC3 I to LC3 II produced by PBLE was effectively blocked by 3MA and wortmannin treatment. Chloroquine (CQ), an inhibitor of autophagy flow, was additionally employed to support the notion that PBLE regulates the autophagy flux. As anticipated, PBLE and CQ therapy significantly accelerated the conversion of LC3 I to LC3 II and the production of p62 protein (Figure 6C). In addition, 3MA and CQ were used in conjunction with PBLE to analyze the Tyr protein level and melanin formation in MNT-1 cells in an effort to ascertain if PBLE-activated autophagy can control melanin biosynthesis. According to Figure 6D,E, both 3MA and CQ treatment restore the PBLE-induced decrease in Tyr protein levels. Similarly to the Tyr result, both inhibitors also restore the melanin production that PBLE had suppressed in MNT-1 cells. These results suggest a connection between autophagy activation and the depigmenting effects of PBLE.

### 3.9. Depigmenting Effects of PBLE on UVB-Induced Pigmented HRM-2 Hairless Mice

**Figure 7:**
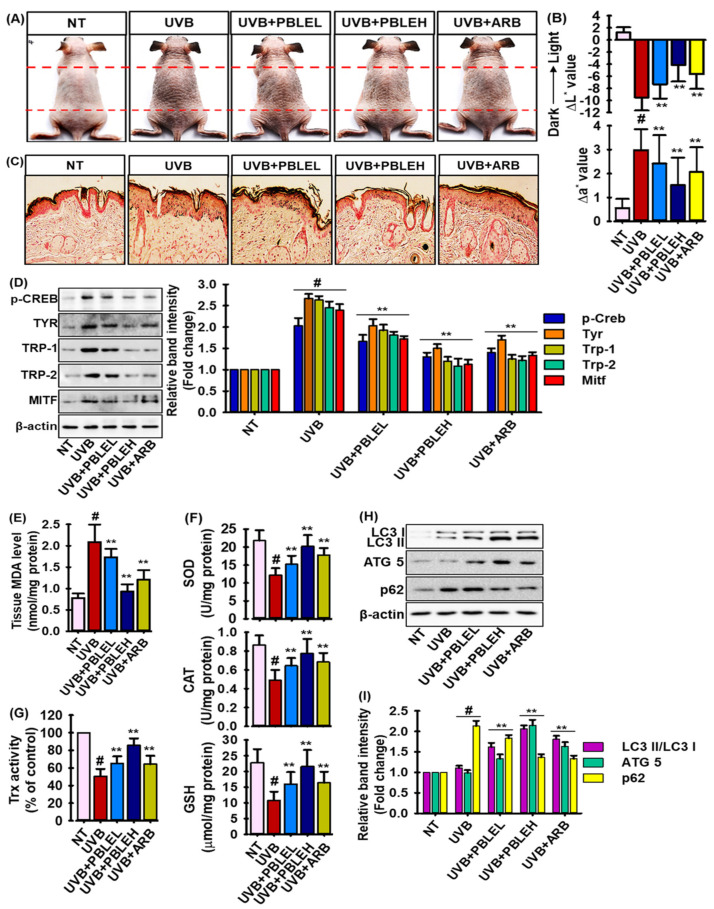
The depigmenting effects of PBLE on UVB-induced HRM-2 mice. (**A**) Representative images demonstrate the dorsal skin’s variations in pigmentation in the studied animals. (**B**) The average values following the final UVB exposure were subtracted from the average baseline values before treatment to determine the ΔL* and Δa* values displayed in the upper and lower panels, respectively. (**C**) The dorsal skin slices of mice stained with Fontana–Masson dye. (**D**) Western blot analysis of p-CREB, tyr, Trp-1, Trp-2, and MITF. Quantification and statistical analysis of the band intensities are in adjacent figure. (**E**) Tissue malonaldehyde (MDA) levels. (**F**) Antioxidant enzymes including superoxide dismutase (SOD), catalase (CAT), and glutathione (GSH) levels in mice skin tissue. (**G**) Tissue thioredoxin activity. (**H**) Autophagy biomarkers such as LC3, Atg5 and p62 were confirmed by Western blot analysis. (**I**) Quantification and statistical analysis of the band intensities of represented Figure 7G. NNFE. Results are presented as the means ± SDs (n =3). # *p* < 0.05 vs. non-treated (NT) control. ** *p* < 0.05 vs. UVB-treated control. ARB: arbutin.

Melanin-producing hairless mice were used to test the inhibitory effects of PBLE on skin pigmentation in vivo. The treatment schedule is depicted in the Supplementary Materials Figure S1. Mice were treated with the vehicle (control group), a PBLE-low (PBLEL) and a PBLE-high dose (PBLEH), or arbutin (positive control), for 3 days before UVB exposure. Mice developed noticeable pigmentation after repeated UVB exposure (Figure 7A). A spectrophotometer was used to measure the skin color. The L* levels dropped after UVB exposure, indicating more pigmentation. The substantial depigmenting effects of PBLE are demonstrated by the dose-dependent attenuation of the UVB-induced fall in L* values in the PBLE-treated animals (Figure 7B, top graph). Additionally, UVB exposure increased a* values, which are a good indicator of sunburns. However, the PBLE concentration-dependently reduced this UVB-induced rise in a* values. Indeed, compared to ARB, PBLE at higher doses had significant depigmenting effects (Figure 7B). The melanin-staining technique Fontana–Masson staining was used to confirm the results of PBLE. Figure 7C shows that animals treated with PBLE showed less melanin staining than the control animals after UVB light, which is consistent with the results of the spectrum. Additionally, PBLE treatment significantly reduced the expression of proteins linked to melanogenesis, such as Tyr, Trp-1, Trp-2, p-CREB, and MITF, compared to the UVB-irradiated controls (Figure 7D). Higher doses of PBLE showed far more significant Tyr and MITF downregulating effects than ARB (Figure 7D). These findings imply that PBLE can reduce UVB-induced skin pigmentation in mice, ostensibly through inhibiting melanogenesis.

Furthermore, UVB-induced oxidative stress (OS) boosts the melanogenesis and antioxidant defense systems and plays a pivotal role in maintaining an optimal redox balance, protecting against OS, excessive melanogenesis, and photo-damaged skin [34]. Thus, to scrutinize whether PBLE boosts the antioxidant defense systems in UVB-induced pigmented mice, malondialdehyde (MDA; lipid peroxidation marker), and various antioxidant enzymes, such as superoxide dismutase (SOD), catalase (CAT), glutathione (GSH) and thioredoxin (Trx), activities were measured. As shown in Figure 7E, UVB treatment significantly boosted the MDA level (2-fold) than the non-treated control. In contrast, PBLE treatment dose-dependently mitigated the MDA level in a significant manner, and at higher doses of PBLE, demonstrated greater lipid peroxidation inhibition than ARB. In addition, the levels of SOD, CAT, GSH, and Trx were significantly downregulated by UVB irradiation, while PBLE treatment reversed this trend in a dose-dependent manner (Figure 7F,G). Interestingly, higher doses of PBLE, as opposed to ARB, strongly augmented endogenous antioxidant enzymes (Figure 7F,G). Moreover, to confirm the autophagic activation by PBLE in UVB-induced pigmenting mice, expressions of autophagic markers including LC3, Atg5, and p62 were evaluated by immunoblotting assay. As described in Figure 7H,I, PBLE treatment augmented the expression of LC3 and Atg5, and suppressed the UVB-induced overexpression of p62, confirming that PBLE also activated the autophagy in vivo. It is noteworthy that PBLEH has greater potential for autophagic activation than ARB.

## 4. Discussion

Melanin production is one of the most important factors influencing the skin and hair color of humans. Melanogenesis involves the production and transport of melanin as well as the release of melanosomes [26]. Several biological compounds, including hydroquinone, have been discovered to treat hyperpigmentation and discoloration of the skin. However, the administration of regularly used whitening therapies with potent Tyr inhibitor, is restricted due to serious side effects such as skin redness, peeling, and vitiligo [35]. Melanin production inhibitors are still being studied by medicinal chemists as a potential treatment for hyperpigmentation issues such melasma, freckles, and age spots. Natural substances, on the other hand, are now recognized as ideal in cosmetic research and development for generating harmless and effective skin whitening agents due to their safety and lack of adverse effects. Notably, betel leaf extract has been utilized for decades to treat skin and eye disorders [24], whereas research on the depigmenting effects of PBLE and its underlying molecular mechanism is scarce. In this study, in vitro and in vivo studies were used for the first time to evaluate the depigmenting effect of betel leaf extract and its underlying mechanism.

Tyrosinase transforms L-tyrosine to L-DOPA (monophenolase) and oxidizes L-DOPA to dopachrome (diphenolase) during melanin production; therefore, mushroom tyrosinase is commonly employed as a target enzyme for the screening of putative inhibitors of melanogenesis [36]. In the existing investigation, PBLE dose-dependently inhibited the tyrosinase activity of mushroom, mice, and human tyrosinase (Figure 2B). In addition, kinetic studies demonstrated that PBLE inhibited the monophenolase activity of mushroom tyrosinase (Supplementary Materials Figure S1). PBLE (100 μg/mL) elongated the lag phase by 15 min compared to the control, corroborating previous findings indicating that licorice root and *Nymphaea nouchali* flower can lengthen the lag phase [4]. Notably, the reaction lag time is influenced by the concentrations of both the enzyme and the substrate, and the presence of *O*-diphenols or transition metal ions can reduce or completely remove the reaction lag time [36]. Additionally, the compounds quercetin, phenylpropanoids, chlorogenic acid, and hydroxycinnamic acid (coumaric, caffeic, and ferulic acid) have the ability to lengthen the lag phase of tyrosinase’s monophenolase activity [36,37,38]. Previous studies revealed that PBLEs are abundant in hydroxycinnamic acid, phenylpropanoids, and flavonoids, which may plausibly play the essential role in extending the lag phase of monophenolase activity of tyrosinase; nevertheless, the study of the specific mechanism is still ongoing.

The primary mechanism by which most Tyr inhibitors limit melanogenesis is the reduction of the numerous melanogenesis-associated proteins, including Tyr, Trp-1, and -2 [4,26]. PBLE drastically lowered the expression of MITF, which is strictly associated with the process of melanogenesis and MITF-dependent enzymes, such as Tyr, Trp-1, and -2, in in vitro and in vivo (Figure 3 and Figure 7). The preceding findings suggest that PBLE can inhibit melanogenesis via inhibiting the expression of Tyr, Trp-1, and -2 through the inactivation of MITF. Recent research indicates that phenolic acid (caffeic, coumaric, and coumaroylquinic acid) [38,39], flavonoids (quercetin, catechin, epigallocatechin, isovitexin, and rutin) [40,41], and coumarins (daphnetin and umbelliprenin) [42] can effectively inhibit melanin synthesis, particularly via the downregulation of Tyr in melanocytes cell culture systems. PBLE are rich in phenolic acid, flavonoids, and coumarins, which could potentially affect their anti-melanogenic action. Consequently, quantification and analyses of the links between the structure and activity of the identified phytochemicals are required to demonstrate their involvement in the anti-melanogenic process in PBLE. In addition, GC–MS study revealed that PBLE had a high concentration of phenylpropanoid (3-allyl-6-methoxyphenol) in its acetate form (3-allyl-6-methoxyphenyl acetate), despite the fact that the significance of these molecules in melanogenesis remains unknown.

IBMX, a cAMP-inducing substance, influences the PKA signaling pathway, which affects melanogenesis as well. Tyr, Trp-1, and -2 are melanogenesis regulators that are expressed by transcription factors such as MITF and cAMP-responsive element-binding proteins (CREBs), which are controlled by the PKA signaling pathway [4]. In this study, IBMX administration dramatically increased intracellular cAMP levels, phosphorylation of CREB, MITF, Tyr, Trp-1 and -2, and melanin synthesis in MNT-1 cells, but PBLE treatment reversed this tendency in a concentration-dependent manner (Figure 5). Prior studies indicated that caffeic acid (IC_50_: 1.9 mM), coumaric acid (IC_50_: 5.7 mM), and ferulic acid (IC_50_: 4.8 mM) prevent the synthesis of melanin in IBMX-induced B16F10 cells through inhibiting the cAMP-MITF pathway [43]. In addition, quercetin, isovitexin, and their glycosides have the ability to suppress melanin formation in B16F10 melanoma cells treated with α-MSH [41]. Additionally, catechin and its derivatives from *Camellia sinensis* and daphnetin-rich *Daphne odora* extract suppressed the cAMP/CREB/MITF signaling cascade to limit the formation of melanin in B16F10 cells [40,42,43]. Therefore, PBLE may possess a potent anti-melanogenesis activity due to the inclusion of many melanogenesis inhibitors; however, it is unclear whether the constituents exert the anti-melanogenic impact singularly or collectively.

There is growing evidence that the MAPKs (ERKs, p38, and JNKs) play important regulating roles in melanogenesis. According to scientific studies, the phosphorylation of ERK, JNK, and p38 by melanogenesis inhibitors leads to the phosphorylation of MITF at serine 73 and the concomitant ubiquitin-dependent proteasomal degradation [4,26]. In this investigation, PBLE phosphorylates ERK, JNK, and p38 in a concentration-dependent manner (Figure 3A), while co-treatment with a JNK and p38 inhibitor significantly reversed PBLEs effects on melanin formation, MITF inhibition, and Tyr inhibition, although an ERK inhibitor had no such impact (Figure 3B,C). This is consistent with our previous report, which stated that the *Nymphaea nouchali* flower, sesamol (lignan of *Sesamum indicum*), and *Euryale ferox* seed inhibited melanin production through the phosphorylation of MAPKs [4,26,44,45].

Several circumstances, including starvation, oxidative stress, and hypoxia, can regulate autophagy. Research has demonstrated that autophagy plays a significant role in melanin metabolism and that it controls the synthesis, maturation, and demise of melanosomes [46]. Nevertheless, it remains unknown how melanosomes are destroyed via autophagy. A recent study has demonstrated that autophagy inducers or alternative autophagy-inducing pathways (autophagy flux) have triggered the autophagic degradation of melanosomes in melanocytes [46]. Myosin-Va knockout (M-KD) cells stimulated by theophylline showed lower melanin, Tyr expression levels, and p62, while increased LC3 II, suggesting that extra melanosomes may be destroyed by autophagy in cells [47]. Autophagy inducers (β-mangostin, resveratrol, 3′-hydroxydaidzein, and ARP101) decrease melanogenesis induced by α-MSH, lower the expression of melanogenesis-related proteins and enzymes (MITF, Tyr, Trp-1, and -2), and destroy melanosomes via inducing autophagosome formation [48,49,50,51]. The melanin-decreasing effects of autophagy inducers were also demonstrated to be inhibited by chloroquine (autophagy flux inhibitor) [14]. In this study, PBLE treatment boosted p62 protein degradation as well as the protein expression of ATG5 and Beclin 1 and the conversion of LC3 I to LC3 II, in both MNT-1 cells and UVB-induced pigmentation of HRM-2 mice (Figure 6 and Figure 7). Interestingly, ARB-treated animals activate autophagy to a lesser level than PBLEH-treated mice (Figure 6). Numerous studies have demonstrated that ARB reduces inflammation, oxidative stress, and apoptosis in human retinal pigment epithelial (ARPE-19) cells, and LPS induced myocardial injury by activating autophagy through the enhancement of the silent mating type information regulation 2 homolog (SIRT1) [52,53]. In addition, Lv et al. [54] demonstrated that ARB activated autophagy to protect HK-2 cells from high glucose-induced apoptosis via upregulating microRNA-27a. Additionally, the melanogenesis-inhibiting effects of PBLE were abolished by the autophagy inhibitors 3MA and CQ in MNT-1 cells (Figure 6). The aforementioned data indicate that PBLE serves as an autophagy inducer, which may be largely responsible for its depigmenting effect. To distinguish between autophagy inducers (which result in pigment degradation) and autophagy-related regulators (which affect pigment synthesis), detailed molecular mechanistic research is still necessary, either by knocking down genes associated with autophagy or by measuring autophagy in the skin of hyperpigmented patients.

UVR can directly worsen melanin production by causing melanocyte proliferation and activation, increasing the number of melanosomes in each melanocyte, and speeding up the transfer of the melanosomes from melanocytes to keratinocytes [34]. Furthermore, the process of melanogenesis causes the generation of reactive oxygen species (ROS), and the existence of oxidative stress (OS) has been anticipated as a pathogenetic explanation for hyperpigmentation. Recent studies have revealed that increases in OS boost Tyr activity, resulting in an exacerbating melanogenesis [6]. In addition, lipid peroxidation is also a significant factor in melanogenesis [6]. The antioxidant defense mechanisms are essential for preserving an ideal redox balance in melanocytes by squelching ROS, defending against oxidative stress, excessive melanogenesis, and photo-damaged skin [34]. The enzymes superoxide dismutase (SOD), catalase (CAT), glutathione (GSH), and thioredoxin (Trx) are among the melanocytes’ endogenous antioxidants [55]. The first-line antioxidant enzymes of defense are SOD, CAT, and GSH, and changes in their activity have an impact on melanogenesis [56,57]. In this study, PBLE significantly suppressed the lipid peroxidation as well as successfully augmented the enzymatic activities of SOD, CAT, and GSH in UVB-induced pigmented HRM-2 mice. In contrast to ARB, mice treated with PBLEH exhibited greater effectiveness to inhibit lipid peroxidation and an increase in antioxidant enzymes. Tang et al. [58] discovered that ARB had the capacity to reduce oxidative stress in retinal pigment epithelial cells via regulating the SIRT1/FOXO3a/PGC-1/pathway. It has also been shown that thioredoxin (Trx) inhibits tyrosinase activity by reversibly interacting with the binuclear copper center of tyrosinase to decrease melanin production. Here, UVB irradiation reduced the Trx activity, while PBLE significantly increased the Trx activity, suggesting that the depigmenting effect of PBLE could also be regulated by boosting the antioxidant defense system. However, whether mitochondrial or peroxisomal antioxidant systems are triggered by PBLE and how they regulate melanogenesis remains unknown.

The depigmenting effects of PBLE on human skin are currently unknown. Besides ethical issues, animal skin only mimics human skin to a limited extent, making it chal-lenging to translate basic and preclinical research into therapeutic practice. These dispari-ties underscore the need for more accurate and representative models. However, the use of animals in dermatological research has increased dramatically in recent years, allowing scientists to understand better the pathophysiological mechanisms underlying skin dis-ease and test potential preclinical therapies [59]. The dermis and epidermis layers of mouse and human skin are the same, although their thickness and quantity are consid-erably different, and their protein-coding gene sequences are only 70% equivalent [60]. Consequently, the need for skin models, which have become an essential tool for re-searching skin physiology and disease, is a limitation of our work.

## 5. Conclusions

**Figure 8:**
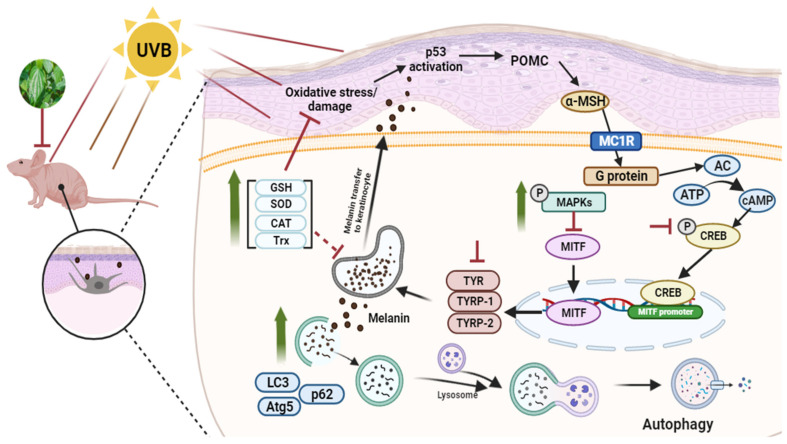
The potentially effective depigmenting mechanism of betel leaves (*Piper betle* L.). Green arrows: activation by PBLE; red arrows: inhibition by PBLE.

In the present study, the inhibitory effects of PBLE on melanin biosynthesis were characterized in MNT-1 cells and the HRM-2 hairless mouse model. The following succinct statement summarizes the significance of this study, as shown in Figure 8: (a) PBLE had strong antioxidant properties and potently repressed tyrosinase activity, followed by melanogenesis. (b) The mechanisms underlying the depigmentation effects of PBLE involved interfering with melanogenesis-related transcription factors and signaling pathways. PBLE caused depigmentation in vitro and in vivo by suppressing cAMP, decreasing CREB phosphorylation, increasing p38 and JNK phosphorylation, and downregulating MITF expression and Tyr, Trp-1, and -2 levels. (c) In addition, recent findings suggest that the observed depigmenting effects of PBLE may result from an augmentation of autophagy and endogenous antioxidant enzymes in vitro and in vivo. Based on our findings and the fact that PBLE did not demonstrate cytotoxicity, we propose that PBLE can be utilized as a potent natural depigmenting agent or composition in food industry as well as cosmetic industry. However, further study is still required to determine the depigmenting effects of PBLE on human skin.

## Figures and Tables

**Table 1:** List of identified compounds of various extract/fractions of PBL by GC–MS.

No.	RT	CAS	Name	MF	MW	Area (%)			
						PBLE	PBLH	PBLA	PBLW
1	22.43	000470-82-6	Eucalyptol	C_10_H_18_O	154.25	0.01	1.21	0.81	Nd
2	25.27	000694-74-6	6-Amino-6-methylfulvene	C_7_H_9_N	107.15	0.01	0.51	1.01	Nd
3	40.47	003856-25-5	α-Copaene	C_15_H_24_	204.35	0.95	1.52	1.84	0.01
4	42.11	005208-59-3	β-Bourbonene	C_15_H_24_	204.35	0.40	0.51	0.74	0.03
5	44.00	000078-70-6	Linalool	C_10_H_18_O	154.25	0.13	1.13	1.53	0.03
6	45.15	013474-59-4	trans-α-Bergamotene	C_15_H_24_	204.35	0.09	1.09	2.09	0.09
7	46.81	000087-44-5	trans-Caryophyllene	C_15_H_24_	204.35	1.80	1.35	1.48	0.03
8	49.65	025246-27-9	Allo-aromadendrene	C_15_H_24_	204.35	0.04	2.14	Nd	Nd
9	50.99	006753-98-6	α-Humulene	C_15_H_24_	204.35	0.83	1.40	1.59	0.07
10	51.87	028973-97-9	cis-β-Farnesene	C_15_H_24_	204.35	0.08	2.08	1.48	Nd
11	52.59	178737-43-4	cis-4,10-epoxy-Amorphane	C_15_H_26_O	222.37	0.03	2.03	1.03	0.03
12	53.23	023986-74-5	Germacrene D	C_15_H_24_	204.35	0.19	0.59	1.19	0.09
13	53.53	157477-72-0	cis-Muurola-diene	C_15_H_24_	204.35	0.12	0.42	1.12	0.02
14	53.64	030021-74-0	γ-Muurolene	C_15_H_24_	204.35	0.03	0.93	1.03	Nd
15	54.11	031983-22-9	α-Muurolene	C_15_H_24_	204.35	0.48	0.02	1.05	Nd
16	54.24	000495-61-4	β-Bisabolene	C_15_H_24_	204.35	0.26	1.26	0.86	Nd
17	57.27	029837-12-5	Cubenene	C_15_H_24_	204.35	0.11	2.81	1.11	0.01
18	60.95	000127-41-3	α-Ionone	C_13_H_20_O	192.30	0.03	3.13	2.93	Nd
19	62.31	000100-51-6	Benzyl alcohol	C_7_H_8_O	108.14	0.02	1.32	4.62	1.92
20	63.47	001198-37-4	2,4-Dimethylquinoline	C_11_H_11_N	157.21	0.03	Nd	1.03	0.03
21	64.13	000060-12-8	2-Phenylethanol	C_8_H_10_O	122.07	0.02	0.82	3.12	1.02
22	64.30	021391-99-1	α-Calacorene	C_15_H_20_	200.32	0.06	0.56	1.06	0.06
23	65.52	000079-77-6	trans-β-Ionone	C_13_H_20_O	192.30	0.02	3.02	2.32	Nd
24	69.12	000093-15-2	Methyl eugenol	C_11_H_14_O_2_	178.23	0.73	0.84	2.54	0.08
25	69.38	002153-66-4	Santrolina triene	C_10_H_16_	136.24	0.04	0.04	Nd	Nd
26	71.48	020129-39-9	α-Corocalene	C_15_H_20_	200.32	0.06	1.26	2.06	0.06
27	72.15	000102-76-1	Triacetin	C_9_H_14_O_6_	218.20	0.09	0.89	0.19	0.09
28	72.33	022567-17-5	γ-Gurjunene	C_15_H_24_	204.35	0.02	0.12	0.08	0.02
29	74.56	077171-55-2	(-)-Spathulenol	C_15_H_24_O	220.35	0.51	0.83	0.82	0.02
30	76.39	000097-53-0	Eugenol	C_10_H_12_O_2_	164.20	0.71	0.52	9.73	0.27
31	77.68	000501-19-9	*O*-Eugenol	C_10_H_12_O_2_	164.20	51.47	26.01	35.25	0.30
32	80.62	001941-09-9	*O*-Eugenol acetate	C_12_H_14_O_3_	206.24	17.37	16.06	26.72	0.20
33	82.56	092691-77-5	Gossonorol	C_15_H_22_O	218.33	0.06	0.76	0.06	Nd
34	83.94	000501-92-8	Chavicol	C_9_H_10_O	134.17	2.01	2.44	3.96	0.25
35	84.17	997146-73-2	Dihydro actinidiolide	C_11_H_16_O_2_	180.24	0.04	0.02	0.04	0.04
36	90.04	000480-33-1	(-)-Mellein	C_10_H_10_O_3_	178.18	0.05	0.15	0.05	0.05

RT: retention time; CAS: chemical abstract service; MF: molecular formula; MW: molecular weight; and Nd: not detected.

## Data Availability

Data are contained within the article and Supplementary Materials.

## Supplementary Materials

The following supporting information can be downloaded at: https://www.mdpi.com/article/10.3390/antiox12020374/s1. Figure S1: experimental schedule for conducting the depigmenting effect of PBLE on UVB-induced pigmentation of HRM-2 mice; Figure S2: effects of PBLE on the monophenolase activity of tyrosinase.; and Table S1: list of antibodies used in this study.

## References

[1] D’Mello S.A., Finlay G.J., Baguley B.C., Askarian-Amiri M.E. (2016). Signaling pathways in melanogenesis. Int. J. Mol. Sci..

[2] Wang N., Hebert D.N. (2006). Tyrosinase maturation through the mammalian secretory pathway: Bringing color to life. Pigment. Cell Res..

[3] Ebanks J.P., Wickett R.R., Boissy R.E. (2009). Mechanisms regulating skin pigmentation: The rise and fall of complexion coloration. Int. J. Mol. Sci..

[4] Alam M.B., Ahmed A., Motin M.A., Kim S., Lee S.H. (2018). Attenuation of melanogenesis by Nymphaea nouchali (Burm. f) flower extract through the regulation of cAMP/CREB/MAPKs/MITF and proteasomal degradation of tyrosinase. Sci. Rep..

[5] Miyamura Y., Coelho S.G., Wolber R., Miller S.A., Wakamatsu K., Zmudzka B.Z., Ito S., Smuda C., Passeron T., Choi W. (2007). Regulation of human skin pigmentation and responses to ultraviolet radiation. Pigment. Cell Res..

[6] Kim Y.J., Yokozawa T. (2009). Modulation of oxidative stress and melanogenesis by proanthocyanidins. Biol. Pharm. Bull..

[7] Jiménez-Cervantes C., Martínez-Esparza M.A., Pérez C., Daum N., Solano F., García-Borrón J.C. (2001). Inhibition of melanogenesis in response to oxidative stress: Transient downregulation of melanocyte differentiation markers and possible involvement of microphthalmia transcription factor. J. Cell Sci..

[8] Kamiński K., Kazimierczak U., Kolenda T. (2022). Oxidative stress in melanogenesis and melanoma development. Contemp. Oncol..

[9] Kondo T., Hearing V.J. (2011). Update on the regulation of mammalian melanocyte function and skin pigmentation. Expert Rev. Dermatol..

[10] Okamoto K. (2014). Organellophagy: Eliminating cellular building blocks via selective autophagy. J. Cell Biol..

[11] Stolz A., Ernst A., Dikic I. (2014). Cargo recognition and trafficking in selective autophagy. Nat. Cell Biol..

[12] Yang Z., Klionsky D.J. (2010). Mammalian autophagy: Core molecular machinery and signaling regulation. Curr. Opin. Cell Biol..

[13] Ganesan A.K., Ho H., Bodemann B., Petersen S., Aruri J., Koshy S., Richardson Z., Le L.Q., Krasieva T., Roth M.G. (2008). Genome-wide siRNA-based functional genomics of pigmentation identifies novel genes and pathways that impact melanogenesis in human cells. PLoS Genet..

[14] Ho H., Ganesan A.K. (2011). The pleiotropic roles of autophagy regulators in melanogenesis. Pigment. Cell Melanoma Res..

[15] Kumar N., Misra P., Dube A., Bhattacharya S., Dikshit M., Ranade S. (2010). Piper betle Linn. a maligned Pan-Asiatic plant with an array of pharmacological activities and prospects for drug discovery. Curr. Sci..

[16] Pradhan D., Suri K., Pradhan D., Biswasroy P. (2013). Golden heart of the nature: *Piper betle* L.. J. Pharmacogn. Phytochem..

[17] Choudhary D., Kale R.K. (2002). Antioxidant and non-toxic properties of Piper betle leaf extract: In vitro and in vivo studies. Phytotherapy Research: An International Journal Devoted to Pharmacological and Toxicological Evaluation of Natural Product Derivatives. Phytother. Res..

[18] Nouri L., Nafchi A.M., Karim A. (2014). Phytochemical, antioxidant, antibacterial, and α-amylase inhibitory properties of different extracts from betel leaves. Ind. Crops Prod..

[19] Majumdar B., Chaudhuri S.G.R., Ray A., Bandyopadhyay S.K. (2003). Effect of ethanol extract of Piper betle Linn leaf on healing of NSAID–induced experimental ulcer-A novel role of free radical scavenging action. Indian J. Exp. Biol..

[20] Parmar V.S., Jain S.C., Gupta S., Talwar S., Rajwanshi V.K., Kumar R., Azim A., Malhotra S., Kumar N., Jain R. (1998). Polyphenols and alkaloids from Piper species. Phytochemistry.

[21] Saravanan R., Prakasam A., Ramesh B., Pugalendi K. (2002). Influence of Piper betle on hepatic marker enzymes and tissue antioxidant status in ethanol-treated Wistar rats. J. Med. Food.

[22] Arambewela L., Arawwawala M., Rajapaksa D. (2006). Piper betle: A potential natural antioxidant. Int. J. Food Sci. Technol..

[23] Atiya A., Sinha B.N., Ranjan Lal U. (2018). New chemical constituents from the *Piper betle* Linn.(Piperaceae). Nat. Prod. Res..

[24] Madhumita M., Guha P., Nag A. (2020). Bio-actives of betel leaf (*Piper betle* L.): A comprehensive review on extraction, isolation, characterization, and biological activity. Phytother. Res..

[25] Alam M.B., Ju M.K., Lee S.H. (2017). DNA Protecting Activities of *Nymphaea nouchali* (Burm. f) Flower Extract Attenuate t-BHP-Induced Oxidative Stress Cell Death through Nrf2-Mediated Induction of Heme Oxygenase-1 Expression by Activating MAP-Kinases. Int. J. Mol. Sci..

[26] Alam M.B., Bajpai V.K., Lee J., Zhao P., Byeon J.H., Ra J.S., Majumder R., Lee J.S., Yoon J.I., Rather I.A. (2017). Inhibition of melanogenesis by jineol from Scolopendra subspinipes mutilans via MAP-Kinase mediated MITF downregulation and the proteasomal degradation of tyrosinase. Sci. Rep..

[27] Górny M., Bilska-Wilkosz A., Iciek M., Hereta M., Kamińska K., Kamińska A., Chwatko G., Rogóż Z., Lorenc-Koci E. (2020). Alterations in the Antioxidant Enzyme Activities in the Neurodevelopmental Rat Model of Schizophrenia Induced by Glutathione Deficiency during Early Postnatal Life. Antioxidants.

[28] Tagrida M., Benjakul S. (2020). Ethanolic extract of Betel (*Piper betle* L.) and Chaphlu (*Piper sarmentosum* Roxb.) dechlorophyllized using sedimentation process: Production, characteristics, and antioxidant activities. J. Food Biochem..

[29] Khorasani Esmaeili A., Mat Taha R., Mohajer S., Banisalam B. (2015). Antioxidant activity and total phenolic and flavonoid content of various solvent extracts from in vivo and in vitro grown *Trifolium pratense* L. (Red Clover). BioMed Res. Int..

[30] Yogeswari S., Bindu K.H., Kamalraj S., Ashokkumar V., Jayabaskaran C. (2020). Antidiabetic, Antithrombin and Cytotoxic bioactive compounds in five cultivars of *Piper betle* L.. Environ. Technol. Innov..

[31] Cabanes J., García-Cánovas F., Lozano J., Garcia-Carmona F. (1987). A kinetic study of the melanization pathway between L-tyrosine and dopachrome. Biochim. Et Biophys. Acta (BBA)-Gen. Subj..

[32] Jung H.J., Bang E., Kim B.M., Jeong S.H., Lee G.H., Chung H.Y. (2019). Loganin Inhibits α-MSH and IBMX-induced Melanogenesis by Suppressing the Expression of Tyrosinase in B16F10 Melanoma Cells. J. Life Sci..

[33] Lee K.W., Kim M., Lee S.H., Kim K.D. (2022). The Function of Autophagy as a Regulator of Melanin Homeostasis. Cells.

[34] Lu Y., Tonissen K.F., Di Trapani G. (2021). Modulating skin colour: Role of the thioredoxin and glutathione systems in regulating melanogenesis. Biosci. Rep..

[35] García-Gavín J., González-Vilas D., Fernández-Redondo V., Toribio J. (2010). Pigmented contact dermatitis due to kojic acid. A paradoxical side effect of a skin lightener. Contact Dermat..

[36] Liu W.-y., Zou C.-m., Hu J.-h., Xu Z.-j., Si L.-q., Liu J.-j., Huang J.-g. (2020). Kinetic Characterization of Tyrosinase-catalyzed Oxidation of Four Polyphenols. Curr. Med. Sci..

[37] Cho S.J., Kwon H.S. (2015). Tyrosinase Inhibitory Activities of Safrole from Myristica fragrans Houtt. J. Appl. Biol. Chem..

[38] Lim J.Y., Ishiguro K., Kubo I. (1999). Tyrosinase inhibitory p-coumaric acid from ginseng leaves. Phytother. Res. PTR.

[39] Ha J.H., Park S.N. (2018). Mechanism underlying inhibitory effect of six dicaffeoylquinic acid isomers on melanogenesis and the computational molecular modeling studies. Bioorganic Med. Chem..

[40] Sato K., Toriyama M. (2009). Depigmenting effect of catechins. Molecules.

[41] Choi M.-H., Shin H.-J. (2016). Anti-melanogenesis effect of quercetin. Cosmetics.

[42] Eom Y.S., Jeong D., Ryu A., Song K.-H., Im D.S., Lee M.-Y. (2022). Daphne odora Exerts Depigmenting Effects via Inhibiting CREB/MITF and Activating AKT/ERK-Signaling Pathways. Curr. Issues Mol. Biol..

[43] Orhan I.E., Deniz F.S.S. (2021). Inhibition of Melanogenesis by Some Well-Known Polyphenolics: A Review. Curr. Pharm. Biotechnol..

[44] Baek S.h., Lee S.H. (2015). Sesamol decreases melanin biosynthesis in melanocyte cells and zebrafish: Possible involvement of MITF via the intracellular cAMP and p38/JNK signalling pathways. Exp. Dermatol..

[45] Baek S.H., Nam I.J., Kwak H.S., Kim K.C., Lee S.H. (2015). Cellular Anti-Melanogenic Effects of a Euryale ferox Seed Extract Ethyl Acetate Fraction via the Lysosomal Degradation Machinery. Int. J. Mol. Sci..

[46] Zhu W., Zhao Z., Cheng B. (2020). The role of autophagy in skin pigmentation. Eur. J. Dermatol. EJD.

[47] Katsuyama Y., Taira N., Yoshioka M., Okano Y., Masaki H. (2017). Disruption of melanosome transport in melanocytes treated with theophylline causes their degradation by autophagy. Biochem. Biophys Res. Commun..

[48] Lee K.W., Ryu H.W., Oh S.S., Park S., Madhi H., Yoo J., Park K.H., Kim K.D. (2017). Depigmentation of α-melanocyte-stimulating hormone-treated melanoma cells by β-mangostin is mediated by selective autophagy. Exp. Derm..

[49] Kim E.S., Chang H., Choi H., Shin J.H., Park S.J., Jo Y.K., Choi E.S., Baek S.Y., Kim B.G., Chang J.W. (2014). Autophagy induced by resveratrol suppresses α-MSH-induced melanogenesis. Exp. Derm..

[50] Kim E.S., Shin J.H., Seok S.H., Kim J.B., Chang H., Park S.J., Jo Y.K., Choi E.S., Park J.S., Yeom M.H. (2013). Autophagy mediates anti-melanogenic activity of 3′-ODI in B16F1 melanoma cells. Biochem. Biophys. Res. Commun..

[51] Kim E.S., Jo Y.K., Park S.J., Chang H., Shin J.H., Choi E.S., Kim J.B., Seok S.H., Kim J.S., Oh J.S. (2013). ARP101 inhibits α-MSH-stimulated melanogenesis by regulation of autophagy in melanocytes. FEBS Lett..

[52] Ma C., Zhang D., Ma Q., Liu Y., Yang Y. (2021). Arbutin inhibits inflammation and apoptosis by enhancing autophagy via SIRT1. Adv. Clin. Exp. Med..

[53] Zhang B., Zeng M., Li B., Wang Y., Kan Y., Wang S., Meng Y., Gao J., Feng W.-s., Zheng X. (2019). Inhibition of oxidative stress and autophagy by arbutin in lipopolysaccharide-induced myocardial injury. Pharmacogn. Mag..

[54] Lv L., Zhang J., Tian F., Li X., Li D., Yu X. (2019). Arbutin protects HK-2 cells against high glucose-induced apoptosis and autophagy by up-regulating microRNA-27a. Artif. Cells Nanomed. Biotechnol..

[55] Peng H.-Y., Lin C.-C., Wang H.-Y., Shih Y., Chou S.-T. (2014). The melanogenesis alteration effects of Achillea millefolium L. essential oil and linalyl acetate: Involvement of oxidative stress and the JNK and ERK signaling pathways in melanoma cells. PLoS ONE.

[56] Li R., Peng A., He C., Wang X., Shi J., Chen L., Wei Y. (2008). Analysis of triptophenolide and its related compounds from Tripterygium wilfordii Hook.f by electrospray ionization tandem mass spectrometry. Int. J. Mass Spectrom..

[57] Delijewski M., Wrześniok D., Otręba M., Beberok A., Buszman E. (2014). Nicotine impact on melanogenesis and antioxidant defense system in HEMn-DP melanocytes. Mol. Cell. Biochem..

[58] Tang H., Du H., Kuang X., Huang H., Zeng J., Long C., Zhu B., Fu L., Wang H., Zhang Q. (2022). Arbutin Protects Retinal Pigment Epithelium Against Oxidative Stress by Modulating SIRT1/FOXO3a/PGC-1α/β Pathway. Front. Genet..

[59] Avci P., Sadasivam M., Gupta A., De Melo W.C.M.A., Huang Y.-Y., Yin R., Chandran R., Kumar R., Otufowora A., Nyame T. (2013). Animal models of skin disease for drug discovery. Expert Opin. Drug Discov..

[60] Ali N., Hosseini M., Vainio S., Taïeb A., Cario-André M., Rezvani H.R. (2015). Skin equivalents: Skin from reconstructions as models to study skin development and diseases. Br. J. Dermatol..

