# Supplementary material for: Antioxidant Potential-Rich Betel Leaves (Piper betle L.) Exert Depigmenting Action by Triggering Autophagy and Downregulating MITF/Tyrosinase In Vitro and In Vivo
Source: Antioxidants (Basel). 2023 Feb 3;12(2):374. doi: 10.3390/antiox12020374 (PMC9952209; doi:10.3390/antiox12020374)
Supplement: Supplementary file 1 [file antioxidants-12-00374-s001.zip › antioxidants-2166703-supplementary.pdf]

<u>**Supplementary data set**</u>

Antioxidant potential-rich Betel leaves (Piper betle L.) exert depigmenting action by triggering autophagy and downregu-lating MITF/Tyrosinase in vitro and in vivo

Md Badrul Alam[1,2,] Na Hyun Park[1], Bo-Rim Song[1] and Sang-Han Lee[1,2,*]

[1]Department of Food Science and Biotechnology, Graduate School, Kyungpook National University, Daegu 41566, Republic of Korea

[2]Food and Bio-Industry Research Institute, Inner Beauty/Antiaging Center, Kyungpook National University, Daegu 41566, Republic of Korea

*Corresponding author:

Dr. Sang-Han Lee; Department of Food Science and Biotechnology, Kyungpook National University, Daegu 41566, Korea, Phone: (82)053-950-7754 (Office); Fax: 053-950-6772; Email: sang@knu.ac.kr

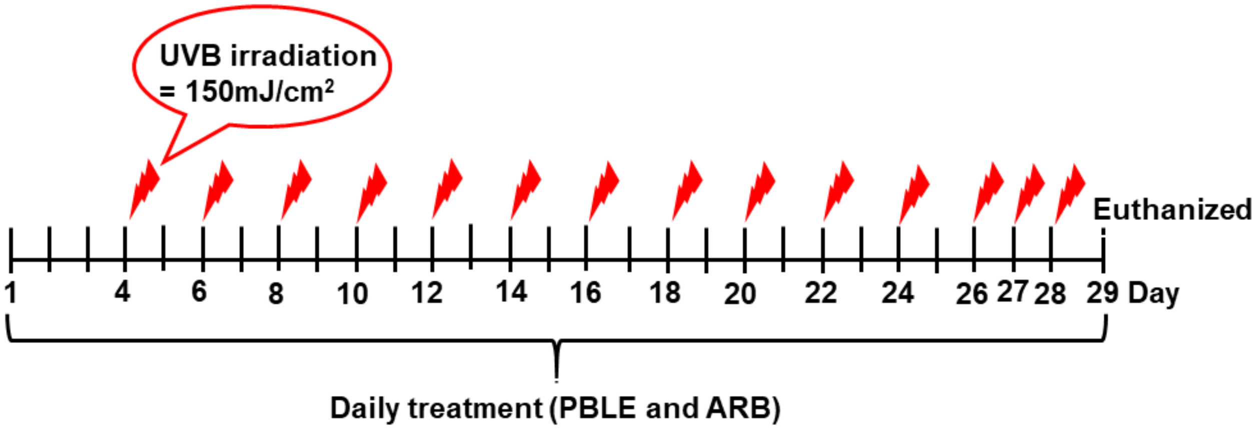

Figure S1. Experimental schedule for conducting the depigmenting effect of PBLE on UVB-induced pigmentation of HRM-2 mice. HRM-2 melanin-possessing hairless mice were treated with the vehicle or PBLE on a designated site on the dorsal skin, according to the indicated schedules. ARB: arbutin.

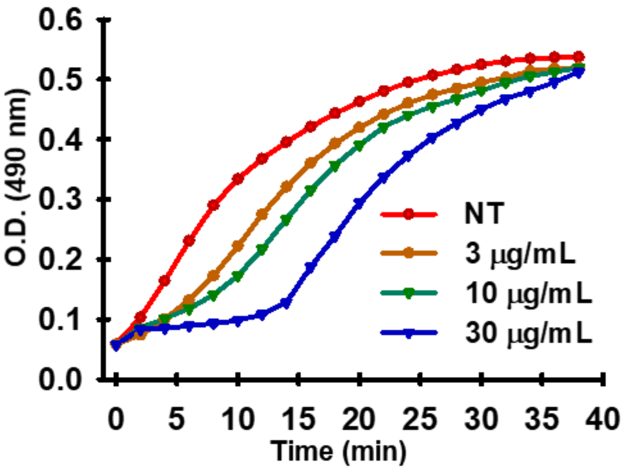

Figure S2. Effects of PBLE on the monophenolase activity of tyrosinase. Enzyme activity was tested in the presence of L-tyrosine, as a substrate. Results are presented as the means ± SDs of three experiments.

Table S1. List of antibodies used in this study.

Antibody	Dilution	Supplier	Catalog No.	Host	MW, kDa
Anti-TYR	1:1000	Abcam, Cambridge, UK	ab170905	Rabit	60
Anti-TRP-1	1:1000	Abcam, Cambridge, UK	ab235447	Rabit	61
Anti-TRP-2	1:1000	Abcam, Cambridge, UK	ab221144	Rabit	59
Anti-MITF	1:1000	Abcam, Cambridge, UK	ab140606	Rabit	58
Anti-p-p38	1:500	Bioworld Technology, Inc.	BS4635	Rabit	42
Anti-p38	1:500	Bioworld Technology, Inc.	BS3567	Rabit	42
Anti-p-ERK1/2	1:500	Bioworld Technology, Inc	BS5016	Rabit	42,44
Anti-ERK1/2	1:500	Bioworld Technology, Inc.	BS 6472	Rabit	42,44
Anti-JNK	1:500	Bioworld Technology, Inc.	BS3630	Rabit	46,54
Anti-p-JNK	1:500	Bioworld Technology, Inc.	BS4322	Rabit	46,54
Anti-CREB	1: 1000	Abcam, Cambridge, UK	ab31387	Rabit	43
Anti-p-CREB	1:1000	Abcam, Cambridge, UK	ab194687	Rabit	43
Anti-LC3	1: 2000	Abcam, Cambridge, UK	ab192890	Rabit	15
Anti-ATG5	1:10000	Abcam, Cambridge, UK	ab108327	Rabit	55
Anti-Beclin1	1: 5000	Abcam, Cambridge, UK	ab207612	Rabit	52
Anti-p62	1:10000	Abcam, Cambridge, UK	ab109012	Rabit	55
Anti-β-actin	1:1000	Abcam, Cambridge, UK	ab8227	Rabit	45
